# Evaluation of the white test effectiveness for the prevention of bile leakage after liver resection: multicenter randomized controlled study

**DOI:** 10.1007/s13304-025-02210-4

**Published:** 2025-04-30

**Authors:** Oleg G. Skipenko, Arkady L. Bedzhanyan, Nikita K. Chardarov, Irina B. Ermak, Andrew D. Ermak, Oleg O. Rummo, Dzmitry A. Fedoruk, Oleg G. Kotenko, Airazat M. Kazaryan

**Affiliations:** 1 Department of Hepatopancreatobiliary Surgery, Petrovsky National Research Center of Surgery, Moscow, Russia; 2 Department of Colorectal Surgery, Petrovsky National Research Centre of Surgery, Moscow, Russia; 3 Department of Hepatopancreatobiliary Surgery, Loginov Moscow Clinical Scientific Center, Moscow, Russia; 4 Department of Liver Transplantation, Shumakov Federal Research Center of Transplantology and Artificial Organs, Moscow, Russia; 5 Department of Hepatopancreatobiliary Surgery, Minsk Scientific and Practical Centre of Surgery, Transplantology and Hematology, Minsk, Belarus; 6 Department of Hepatopancreatobiliary Surgery, Shalimov National Institute of Surgery and Transplantology, Kiev, Ukraine; 7 Department of Gastrointestinal and Pediatric Surgery, Oslo University Hospital - Ullevål, Oslo, Norway; 8 Department of Gastrointestinal Surgery, Østfold Hospital Trust, Grålum, Norway; 9 Department of Faculty Surgery №2, I.M.Sechenov First Moscow State Medical University, Moscow, Russia; 10 Department of Surgery N1, Yerevan State Medical University After M. Heratsi, Yerevan, Armenia

**Keywords:** Bile leakage, Complications, Liver resection, Randomized trial, White test

## Abstract

Bile leakage is a common complication after liver resection. It often requires repeated interventions or surgery and prolongs the patient’s recovery. The aim of the study was to assess the effectiveness of the leakage test with fat emulsion (the White Test) in preventing postoperative biliary complications. A multicenter (3 hospitals) randomized controlled trial was performed from February 2011 to May 2016. The trial involved only the patients scheduled for major hepatectomies. After liver transection and control of biliary tree leak-proofness, the patients were randomized into two groups—with and without applying the White Test. A comparative assessment of all the White Test participants was conducted. Forty-three patients formed the study group, and 36 patients were included in the control group. The White Test revealed sites of bile leakage (the positive White Test) in 37.2% (16/43) of the patients in the study group. These leakage sites were sealed intraoperatively. One of those patients (6.2%; 1/16) still developed bile leakage after surgery. Bile leakage was still observed in 7.4% (2/27) of patients after the negative White test. The incidence of postoperatively revealed bile leakage in the study and control groups did not have a statistically significant difference: 7% (3/43) and 8.3% (3/36), respectively. All bile leaks were grade B. This study demonstrated that the White Test did not provide any benefit in preventing postoperative bile leakage; therefore, other methods, such as ICG, should be further investigated.

## Introduction

Bile leakage is a common complication occurring after hepatobiliary surgery [1]. The incidence of postoperative bile leakage after liver resection ranges from 6.7 to 18.6% [2–5]. The presence of bile in the peritoneal cavity caused by bile leakage may lead to the development of bilomas, internal fistulas, and biliary peritonitis. Bile leakage seriously affects postoperative quality of life and causes intra-abdominal infection and liver failure. In addition, bile leakage significantly prolongs reconvalescence and median postoperative hospital stay [2].

Various methods have been introduced to prevent postoperative bile leakage: intraoperative cholangiography, bile leakage tests, common bile duct drainage, local hemostatics, and omentoplasty. One of them is the White Test, which implies the injection of fat emulsion through the cystic duct after liver resection. The presence of fat emulsion on the transected surface of the liver confirms the leakage that should be eliminated by stitching to avoid postoperative bile leakage [6].

The White Test efficacy was evaluated in the systematic review [7], including three cohort studies [7–9] and one randomized controlled study [10], involving 369 patients. Linke et al. [7] concluded that the White Test reduces the incidence of posthepatectomy biliary complications, but the presented studies’ evidence level was insufficient [11]. Therefore, we performed our own randomized controlled study to assess the impact of the White Test on the incidence of bile leakage after liver resection.

## Materials and methods

### Patients

In the period from February 2011 to May 2016, it was conducted by three centers: the Petrovsky National Research Center of Surgery (Moscow, Russia), the Minsk Scientific and Practical Center of Surgery, Transplantology and Hematology (Minsk, Belarus), and the Shalimov National Institute of Surgery and Transplantology (Kyiv, Ukraine). Patients operated on by the surgeons participating in this project (OGS, ALB, NKC, OOR, DAF, and OGK) in ordinary working hours were available for randomization.

This blind randomized study with an allocation ratio of 1:1 followed those patients of the above-mentioned centers who had undergone major hepatic resections (i.e., resections of three or more segments according to Brisbane classification) [12]. Exclusion criteria were as follows: extrahepatic division of the lobar bile ducts, cholangitis before liver resection, non-radical surgery for alveococcosis, surgical interventions on the extrahepatic bile ducts (hepaticojejunostomy), external duct drainage, and preoperative stenting of biliary ducts.

### Ethical aproval

The Institutional Review Board approved the study 13.05.2010, and it was registered in the institutional trial registry (reference number 13.05.2010/5). Consent was obtained from all patients or patients’ parents included in this study.

### White test randomization and performing

**Fig. 1:**
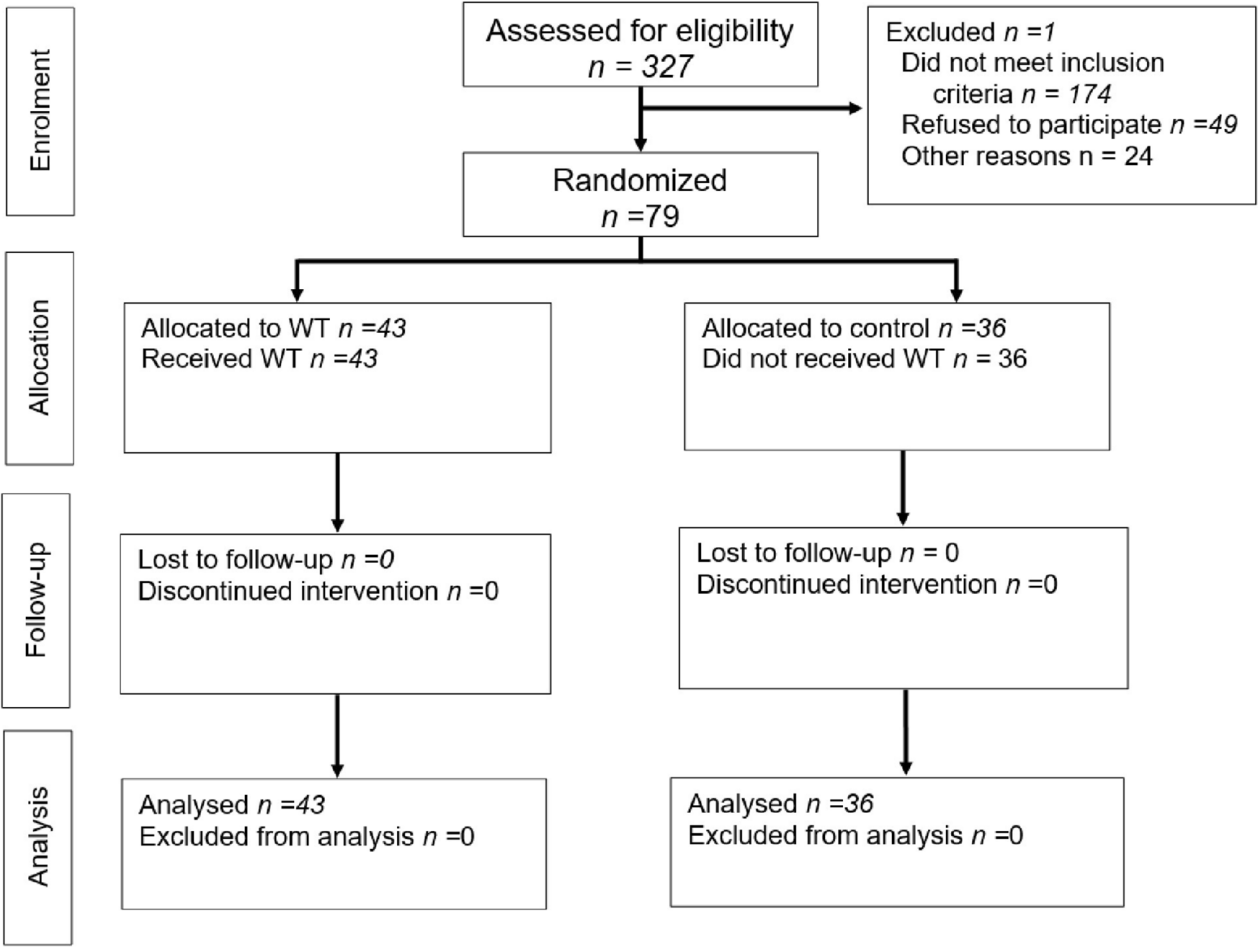
CONSORT flowchart

The study was conducted according to the CONSORT 2010 statement [13]. See CONSORT flowchart (Fig. 1).

**Fig. 2:**
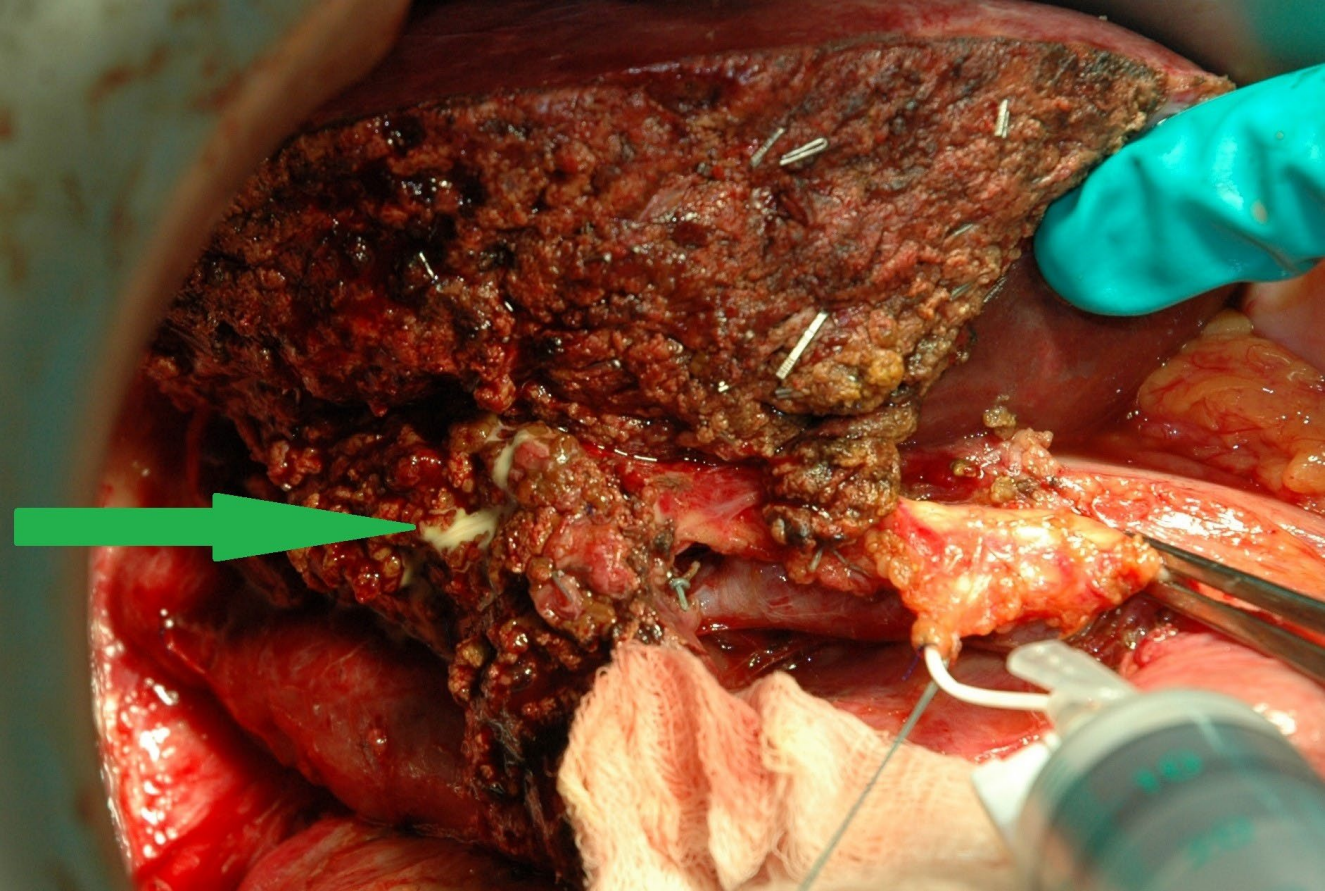
Intraoperative picture of detection of bile leakage by means of the White test (Marked by the green arrow)

After completing the liver resection, the cholestasis was checked by applying white gauze to the liver surface. The visible points of bile leakage were eliminated by ligation/clipping of small ducts or sutures. After the assessment of cholestasis level, randomization based on the method of sealed envelopes was performed. The final stage of the operations in the control group of patients was the drainage of the operative field. The biliary trees of the study group patients were catheterized (through the stump of the lobular or cystic duct), and the common bile duct was clamped in the supraduodenal portion with a “bulldog” clamp. Fat emulsion (Lipofundin® MCT/LCT 10%, B. Braun Melsungen AG, Melsungen, Germany) was injected via the catheter. The filling capacity of the biliary tree was controlled with palpation by the turgor of the common hepatic duct, achieving its moderate tension. The presence of the white fluid was then assessed on the transected liver surface. Detected sites of bile leakage were stitched (Fig. 2). Then, the liver surface was washed with saline solution, and the test was repeated until tightness was achieved. The test was completed by washing out the emulsion from the biliary tree with a saline solution. The test was considered positive when areas of bile leakage were discovered. The number of leaky sites was also taken into account.

The coordinating operative nurse from the Petrovsky National Research Center of Surgery performed the randomization. The responsible operative nurses at the Minsk Scientific and Practical Center of Surgery, Transplantology, and Hematology and at the Shalimov National Institute of Surgery and Transplantology received allocation prescriptions from the coordinating nurse at the Petrovsky National Research Center of Surgery. The sealed envelopes method was applied to generate a random allocation sequence. No blocking was performed. It was patient-blinded, i.e., the patients did not know whether the White Test aimed to diagnose and control possible bile leakage was performed on them or not. Randomization was done intraoperatively immediately after parenchyma transection was done to ensure that the patient fulfilled the criteria for inclusion to randomization (single major resection).

### The postoperative bile leakage diagnosis

All patients received an abdominal drain before the laparotomy closure. The drain was removed routinely on 2 or 3 postoperative days.

The diagnostic criteria of the International Study Group of Liver Surgery (ISGLS) were used to determine biliary complications. In this case, the criteria of postoperative bile leakage are as follows:

1. On the third day after the surgery, the bilirubin concentration in the discharge of drainage exceeds the bilirubin concentration in the rash three or more times; or.
2. Confirmation of intra-abdominal bile accumulation utilizing percutaneous drainage or cholangiography.

Bile leakage severity was classified according to the duration and tactics of treatment. Grade A—does not require treatment or requires minor additional treatment that does not significantly affect the patient’s condition; Grade B—additional diagnostic tests or interventions are necessary, but there is no need for repeated surgery or grade A bile leakage lasting more than 7 days; Grade C—relaparotomy is required to eliminate bile collections, bile peritonitis, and the source of bile leakage [14]. The severity of other complications was determined according to the Dindo–Clavien classification [15].

### Statistical analysis

The power calculation was based on expected differences between postoperative leakage, 3% versus 15% (6, 10). It necessitated including 55 patients in each group in case of considering 60% power with alpha 0.05 and beta 0.4.

All numerical variables are represented as median (minimum and maximum values; ME (min–max). The numerical variables comparison was carried out based on the Mann–Whitney test. The qualitative variables were compared using the *χ*^2^ criterion or Fisher’s exact criterion (if the number of observations in the group is < 5). Differences at *p* < 0.05 were considered statistically significant. Data were analyzed using the Statistica 8.0 package (StatSoft, Tulsa, OK, USA).

## Results

Seventy-nine patients with a median age of 53 (3–79) years were enrolled, including 33 men and 46 women. During randomization, they were assigned to the study (*n* = 43) and control groups (*n* = 36), which did not differ by gender (men: 44.2% and 38.9%, respectively, *p* = 0.63) and age [53 (3–75) and 54 (7–79) years, respectively, *p* = 0.48]. The clinical characteristics of the patients are shown in Tables 1, 2, 3, 4, 5, 6. Colorectal cancer liver metastases were the main indication for liver resection: 40% (32/79). Right hemihepatectomy was performed in 54% (43/79) of cases, and left hemihepatectomy was performed in 41.7% (33/79). Two patients underwent mesohepatectomy, and one—extended right hemihepatectomy. There were no statistically significant differences between the study and control groups regarding indications for liver resection, resection volume, and postoperative complications (*p* > 0.05).

**Table 1:** Indications for surgery

Diagnosis	*n*	Study group *n* = 43	Control group *n* = 36	p
Metastatic colorectal cancer	32	15 (34.9%)	17 (47.2%)	0.26
Hemangioma	12	8 (18.6%)	4 (11.1%)	0.53
Hepatocellular carcinoma	9	6	3	0.49
Echinococcosis	7	4	3	1.0
Adenoma	3	1	2	0.58
Focal nodular hyperplasia	3	3	0	0.24
Cholangiocarcinoma	3	2	1	1.0
Alveococcosis	2	0	2	0.2
Hepatoblastoma	2	1	1	1.0
Cystadenoma	1	0	1	0.45
Hamartoma	1	1	0	1.0
Lymphoma	1	1	0	1.0
Cyst	1	0	1	0.45
Live liver donor	1	1	0	1.0
Metastases of cervical cancer	1	0	1	0.45

**Table 2:** Additional procedures

	n		Study group *n* = 43	Control group *n* = 36
Sg 1 resection		16	10	6
Contralateral lobe segmental wedge resection		7	3	4
Inferior vena cava resection		2	-	2
Right adrenal resection		1	1	-
Radiofrequency ablation of the tumor		1	1	1
Ileostomy closure		1	1	-
Colostomy closure		1	-	1
Colostomy		1	-	1
Total, procedures		31	16	15
Total, patients		28	14	14

**Table 3:** Intraoperative parameters

	*n*	Study group *n* = 43	Control group *n* = 36	*p*
Type of operation				
Right hemihepatectomy	43	24 (55.8%)	19 (52.8%)	0.78
Left hemihepatectomy	33	19 (44.2%)	14 (38.9%)	0.63
Mesohepatectomy	2	0	2	0.20
Extended right hemihepatectomy	1	0	1	0.45
Other intraoperative characteristics				
Blood loss (ml)		400 (100–1800)	350 (100–2000)	0.3
Operative time (min)		270 (150–430)	255 (11–540)	0.17

**Table 4:** Postoperative complications

Postoperative complications	*n*	Study group *n* = 43	Control group *n* = 36	*p*
Complications (n patients)	13	10 (23.3%)	3 (8.3%)	0.12
Complications (n complications)	14	11	3	0.07
**Bile leakage**	**6**	**3 (7%)**	**3 (8.3%)**	**1.0**
Ascites	2	2	–	0.5
Pleural effusion	2	2	–	0.5
Liver failure	1	1	–	1.0
Lymphorrhea	1	1	–	1.0
Hemorrhage	1	1	–	1.0
Wound infection	1	1	–	1.0

**Table 5:** Grading the severity of postoperative complications

	*n*	Study group *n* = 43	Control group *n* = 36	*p*
The severity of complications according to the Dindo–Clavien classification				
Grade I	3	3 Ascites-2 Wound infection-1	–	0.24
Grade II	6	6 Hemorrhage-1 Liver failure-1 Lymphorrhea-1 Bile leakage-3	–	**0.02**
Grade IIIa	5	2 pleural effusion-2	3 Biloma-3	0.65
Grade IIIb		–	–	
The severity of bile leakage				
Bile leakage		3 (7%)	3 (8.3%)	1.0
Grade A		–	–	
Grade B		3 (fistulas)	3 (bilomas)	1.0
Grade C		–	–

**Table 6:** Applied dissection techniques and distribution of bile leakages

Technique	*n*	Study group *n* = 43		Control group *n* = 36	
		*n*	Bile leakage, *n* (%)	*n*	Bile leakage, *n* (%)
CUSA	44	23	1 (4,3)	21	2 (9,5)
Clamp-crushing	28	16	2 (12,5)	12	1 (8,3)
Thunderbeat	3	1	-	2	-
Ligasure	2	1	-	1	-
Harmonic scalpel	2	2	-	-	-

43 patients underwent the White Test. The test revealed sites of bile leakage (positive White test) in 37.2% (16/43) of patients of the study group. From 1 to 2, sources of bile leakage were detected in all cases. The sources identified were as follows: liver transection surface (*n* = 11), extrahepatic ducts (*n* = 2), and lobular duct stump (*n* = 3). All revealed intraoperative bile leakages were sealed intraoperatively. One of the patients with a positive White Test still developed a bile fistula after surgery (6.2%; 1/16). The test was negative in 27 patients (62.8%). Two of them (7.4%; 2/27) had biliary complications after surgery.

The frequency of postoperatively revealed biliary complications in the study and control groups did not differ: 7% (3/43) and 8.3% (3/36), respectively, *p* = 1.0. In all cases, the observed biliary complications were of Grade B severity. In the study group, they were represented by fistulas that functioned for 18, 20, and 35 days. The bile leakage was observed in all patients through drains installed during the operation. The bile leakage in the control group was represented by bilomas, diagnosed by the computed tomography, which required percutaneous drainage. The duration of drains functioning was as follows: 25, 26, and 40 days.

## Discussion

While the incidence of serious hemorrhagic complications and postoperative liver failure decreased significantly in the past 3 decades, the bile leakage rate has been quite stable and has become, at the present time, the leading specific surgical complication after liver resection. The incidence of bile leakage has not changed over the past few years, ranging from 6.7 to 18.6% [2–5]. In most cases, the bile leakage does not threaten the lives of patients. On the other hand, in some cases, the bile leakage causes the development of septic complications that increase the risk of postoperative death. According to Li S. et al., bile leakage significantly increases the length of postoperative hospital stay compared to the patients without biliary complications (52 ± 25 and 28 ± 10 days, respectively, *p* < 0.001) [16]. In addition to the prolongation of hospital stay and reconvalescence period, the bile leakage drives the need for repeated surgery or interventional procedures.

Studies that determine the bile leakage risk factors remain sparse and controversial. It has been noted that biliary complications appear more often after hepatic resection in case of malignancy than after hepatic resection in case of benign disease (19.5% and 4.8%, respectively, *p* < 0.05) [17]. Some types of resections have an increased risk of bile leakage. For instance, mesohepatectomy, bisegmentectomy 5 and 8, segmentectomy 1, and segmentectomy 4 are associated with the increased risk of devascularization and damage of large bile ducts [18, 19]. Operative time, intraoperative blood loss of more than 1.5 l, and the presence of acute purulent inflammation in the preoperative period are also considered as predictors of the bile leakage development [19, 20].

The bile leakage prevention was actively considered even in the 1980 s. Kubo S. et al. were the first to suggest using cholangiography before liver resection to reduce the incidence of postoperative biliary complications [21]. Follow-up studies were aimed at finding an optimal method for intraoperative diagnosis of bile leakage. Several trials have been devoted to bile leakage tests with saline solution. Ijichi M. et al. were among the pioneers in this research field. However, they showed no significant difference in the frequency of bile leakage between the study and control groups (6% and 4%, *p* = 0.95) [22]. Interestingly, Kayaalp C. et al. significantly decreased the rate of bile leakages owing to the test with saline solution (8.8% and 27.7%, respectively, *p* = 0.03). The postoperative hospital stay of the patients who received this procedure was also reduced [23].

Lam C. et al. used methylene blue for leakage testing. There was a decrease in the incidence of postoperative bile leakage in the study group compared with the control group (3.6% and 7.4%, respectively, *p* < 0.05) [24]. Zimmitti G. et al. suggested using the gas test to diagnose the bile leakage. The essence of the method was to introduce gas into the lumen of the biliary tree and visualize it using ultrasound. When the gas bubbles accumulated in the ducts, the bile ducts were supposed to be leakproof. According to the study, using the test significantly improved the intraoperative detection of bile leakage, and the incidence of postoperative biliary complications was significantly higher in the control group (62.1% and 8.3%, *p* < 0.001) [25].

Another method is used for the bile leakage intraoperative diagnosis, which consists of indocyanine green (ICG) introduction into the common bile duct. The disadvantage of this approach is its complexity due to the use of an infrared camera. This test increases the probability of the bile leakage intraoperative detection compared to the saline solution [26]. The study of Kaibori M. et al. reported a reduction of postoperative bile leakage rates after liver resection from 10 to 0% due to intraoperative verification of bile leakages assisted by ICG [27]. Another study conducted by Hanaki T. et al. has similarly reported a reduction of postoperative bile leakage rate from 7.7 to 0% [28]. Both studies were retrospective comparative studies. Surprisingly, the recent systematic review and meta-analysis of ICG application for liver resection for liver malignancies has not found advantages of intraoperative ICG in the prevention of postoperative bile leakages, reporting similar rates of postoperative bile leakages in both groups, 3.1% in ICG group and 3% in non-ICG group [29]. These data are in significant contrast with outcomes reported by Kaibori et al. and Hanaki et al. [27, 28]. Although ICG in liver surgery has spread tremendously and has shown evidence to be effective in improving the rate of R0 liver resection [29], its role in the prevention of postoperative bile leakages should be verified in prospective trials [30].

In this study, we tested the effectiveness of the White Test (the fat emulsion injection through the cystic duct) for the prevention of postoperative biliary complications, proposed by Nadalin S. et al. [6]. Initially, the White Test intraoperative application demonstrated a significant reduction in postoperative bile leakage cases compared to the control group (5% and 22%, *p* < 0.05) [6]. The recent research works results are ambiguous. Only one randomized trial (*n* = 107) regarding the White Test effectiveness evaluation was described in the literature when this work was initiated. In this work, Liu Z. et al. also showed a difference in the frequency of biliary complications between the group with the White Test and the control group (3.7% and 14.8%, *p* < 0.05) [10]. Similar outcomes were obtained in another center, but that research was a cohort study [8].

Interestingly, the White Test has intraoperatively revealed bile leakages in 37.2% of cases. This rate was significantly higher than the postoperative bile leakage rate in our control group (8.3%) and rates reported in the literature [32]. This raises the question of whether many bile leakages diagnosed by the White test could have been ordinarily clinically insignificant and did not actually need management intraoperatively after liver resection. Another hypothesis may attribute the high rate of intraoperative bile leakages diagnosed by the White Test to an induction of increased pressure in the biliary system when carrying out the White Test. These speculative reasons for the high rate of bile leakages revealed by the White test require further specifically designed studies.

The White Test can be less practical in the case of laparoscopic liver resection; nevertheless, the method can be adapted to a minimally invasive surgical technique [33]. However, ICG-compatible equipment for laparoscopic surgery has a clear trend of becoming a standard in high-income countries [34]; thus, the ICG method tends to be more applicable in the laparoscopic setting.

In this study, we did not demonstrate a significant reduction in the frequency of biliary complications after the White Test application. The number of postoperative bile leakage cases did not significantly differ between the group that had undergone the White Test and the control group (7% and 8.3%, *p* > 0.05). The negative White Test and additional preventive measures in case of a positive result did not guarantee the absence of bile leakage in the postoperative period**.** In addition, the operative time was approximately 15 min longer in the White Test group, but this difference was not significant (possibly due to statistical error, type 2).

This study has limitations. The relatively small number of patients included in this study can explain the lack of the White Test’s effectiveness in bile leakage prevention after liver resection. Besides, the study did not recruit the planned number of patients, and it randomly had more patients in the study group than in the control group. These factors and the low incidence of biliary complications in specialized centers may contribute to the possibility of statistical error, type 2. In addition, in recent years, technological improvements in the methods of intraoperative bile leakage control have been made that might potentially improve these methods, including both the White Test and especially ICG methods, which have blossomed tremendously [31, 34]. The present study is reported with a considerable procrastination time frame, which can be considered a limitation too.

## Conclusion

This study demonstrated that the White Test did not provide any benefit in preventing postoperative bile leakage; therefore, other methods, such as ICG, should be further investigated.

## Data Availability

The trial’s data can be made available upon a reasonable request to the corresponding author.
